# The features and factors in the acquisition of English existential constructions at the syntax–pragmatics interface by Chinese learners

**DOI:** 10.3389/fpsyg.2022.983547

**Published:** 2022-08-16

**Authors:** Shan Jiang, Huiping Zhang

**Affiliations:** School of Foreign Languages, Northeast Normal University, Changchun, China

**Keywords:** Interface Hypothesis, existential constructions, syntax–pragmatics interface, Chinese learners, non-native performance

## Abstract

This study adopted a mixed-method study design to investigate the acquisitional features of English existential constructions at the syntax-pragmatics interface by Chinese learners, and explore the factors for non-native performance from the perspective of the Interface Hypothesis. A questionnaire was administered online to 300 Chinese learners of English and 20 English natives at a university in China, which included a picture description test and a context-matching test. Follow-up interviews were conducted with 30 Chinese learners. The experimental data were conducted using comparing means and generalized linear mixed model. Results showed that Chinese learners overproduced existential constructions and reached a native-like level until the advanced stage. Moreover, Chinese learners displayed different preference patterns for existential constructions from English natives, and basically reached a native-like level by the intermediate stage. The qualitative data provided possible explanations for non-native performance. The analysis revealed that non-native performance in production attributed to L1 negative transfer and input frequency, while that in comprehension resulted from underspecification of form-function mapping, input frequency and contexts. Based on the findings, some implications on syntax-pragmatics teaching and L2 interface studies are provided.

## Introduction

In recent years, the Interface Hypothesis (IH; Sorace, 2011) has been proposed to account for non-native performance at the advanced stage of adult L2 acquisition. According to the IH, the syntax-pragmatics interface is a main locus of acquisition delays and processing difficulties even at L2 ultimate attainment. The IH has spurred a fruitful line of research on the acquisition of the syntax-pragmatics interface. Some of them have provided evidence validating syntax-pragmatics vulnerability, while others have challenged the IH (Teixeira, 2020). This raises a question: Are difficulties at the syntax-pragmatics interface global or limited to the particular phenomena which have been chosen for investigation (White, 2011)? In view of this, some scholars claimed that the linguistic phenomena touched upon so far are quite limited, and thus more syntax-pragmatics interface structures are urged to be examined to verify the IH (Jin and Ke, 2021). Given that English existential constructions can only be used if their postverbal noun phrases (NPs) represent hearer-new information in the discourse, they clearly involve the syntax-pragmatics interface and are a good test case for exploring the acquisition of this interface within the framework of the IH.

The acquisition of English existential constructions can be divided into two categories based on research perspectives. It has predominantly been investigated from the perspective of a single dimension regarding syntax and pragmatics as separate, which can be further divided into three subcategories. Some studies in the first subcategory focused on the definiteness restriction on existential constructions, which generally found this restriction is not difficult for L2 learners (White, 2008; White et al., 2012; Rahimi and Youhanaee, 2013). The second subcategory compared the overall usage features of existential constructions between L2 learners and English natives (Palacios-Martínez and Martínez-Insua, 2006; Huang and He, 2007; Yang and Li, 2012; Zhang, 2016; Gong, 2019; Liu and Wang, 2020; Qin and Ding, 2021). The results demonstrated that L2 learners with different L1s exhibited differences in usage frequency and structural complexity from English natives and committed various types of grammatical mistakes. The third subcategory probed into the effect of instruction on the acquisition of existential constructions such as explicit instruction (Youhanaee and Alibabaee, 2009) and task-based instruction (Farsani et al., 2012).

Nonetheless, the acquisition of English existential constructions is an under-researched area from the perspective of the IH. The limited studies focused solely on upper intermediate L2 learners and yielded conflicting findings. By university-student English corpora, some studies investigated the production of English existential constructions at the syntax-pragmatics interface by L2 learners with Italian, Spanish and Greek as L1s (Lozano and Mendikoetxea, 2008, 2010; Agathopoulou, 2014; Mendikoetxea and Lozano, 2018). The results reported that learners produced English existential constructions under the same syntax-pragmatics interface condition as English natives did, that is, producing them only if their postverbal NPs represented new information, but exhibited persistent problems in the syntactic encoding of these constructions. These results revealed that learners’ deficits were not at this interface, but rather syntactic in nature. Teixeira (2020) tested advanced and near-native L2 English learners with L1 Portuguese and L1 French in a battery of timed and untimed tasks. Results showed that, as the IH predicts, both groups exhibited some level of optionality at the syntax-pragmatics interface regarding the types of verbs and discourse contexts compatible with existential constructions. This suggested that optionality is gradient and modulated by four factors: the lower construction frequency, the greater the quantity and/or distance of the contextual information, the greater the difference between L1 and L2, the lower L2 proficiency levels, the more optionality L2 learners tend to display.

Against this backdrop, much remains to be known about problems at L2 syntax-pragmatics interface. Firstly, research participants in the literature have not yet involved Chinese learners who possess various backgrounds and characteristics. As Chinese learners are from different regions in an extremely large country, there are huge individual differences among them in dialect, family and language learning backgrounds. Furthermore, they learn English as a foreign language in formal classroom settings which emphasize on grammar rather than the pragmatic appropriateness and lack genuine and adequate contexts of language use. Consequently, they might present unique features in the acquisition of existential constructions. Secondly, scant attention has been paid to cross-sectional comparisons, whereas an examination of developmental trajectory of the syntax-pragmatics interface among learners is fundamental to learning more about how residual optionality develops and results (Gupton and Calderón, 2021). Additionally, there is a need to determine factors that might account for L2 non-native performance at this interface (Antonova-Unlu and Wei, 2020). Finally, pedagogical enlightenment has not been proposed based on research results to promote second language teaching of this interface.

In light of these inadequacies, it’s meaningful to examine the acquisitional features of English existential constructions at the syntax-pragmatics interface by Chinese learners with different levels, by performing cross-sectional comparisons in production and comprehension. In addition, this study explores the factors for non-native performance within the framework of the IH. Based on this, it may empirically enrich the research on the acquisition of syntax-pragmatics interface constructions, and deepen our understanding of Chinese learners’ learning mechanism from the perspective of syntax-pragmatics interaction. Furthermore, it identifies the difficult locus in the acquisition of English syntax-pragmatics interface constructions, which will be informative for the teaching and learning of English to avoid non-native performance. Specifically, it addresses two research questions:

1. What are the acquisitional features of English existential constructions at the syntax-pragmatics interface in production and comprehension by Chinese learners?
2. What are the factors for non-native performance in production and comprehension by Chinese learners?

## Interface Hypothesis

As the syntax-pragmatics interface involves the integration of syntactic properties and pragmatic conditions, it may result in mapping difficulties or different mappings between interlanguage and native grammars. Sorace (2011) formalized this observation in the Interface Hypothesis, arguing that the syntax-pragmatics interface is the most problematic and vulnerable in L2 acquisition even at the ultimate attainment. Specifically, the IH aims to contend that the syntax-pragmatics interface is a main source of non-native performance in L2 learners, represented in the form of residual optionality, indeterminacy and lasting L1 influence, etc. Unlike many of the developmental problems that are reduced or eliminated as L2 proficiency grows, performance at the syntax-pragmatics interface may remain permanently unstable (Sorace, 2012).

The main challenge raised by the IH is the exploration of reasons for interface vulnerability. Sorace and Serratrice (2009) proposed five factors that contribute to interface vulnerability based on the nature of the syntax-pragmatics interface, and they focused on the first four factors which are psycholinguistic determinants, rather than the fifth factor which is general cognitive. These factors are as follows: (1) underspecification of interface mappings. If a syntax-pragmatics interface mapping of a syntactic structure in L2 is specified, but L1 lacks a similar mapping, the L2 interface mapping will remain underspecified. This underspecification allows a wider range of possible mappings, giving rise to ambiguity and optionality in L2; (2) cross-linguistic influence in representations and/or in parsing strategies. Bilingual speakers’ knowledge representations in each language are influenced by the other language. This influence involves syntax-pragmatics interface conditions that differ between L1 and L2. L1 interface conditions may preserve and access in L2 use, which can be the cause of residual optionality in L2 grammars; (3) processing limitations. The processing of syntax-pragmatics interface requires more processing resources. Nonetheless, L2 learners must use cognitive resources to suppress the activation of their L1, and as a result they may have fewer processing resources available and are less efficient at processing this interface; (4) input received by L2 learners in terms of quantity and quality. L2 learners’ language competence is inevitably affected by the way in which language is actually used. The frequency with which a structure is encountered is bound to have an effect on the speed and accuracy with which it is processed. Compared to monolinguals, L2 learners are exposed to and/or use a reduced amount of L2. This is likely to be related to the reduced integration ability, and less efficient and accurate processing; (5) bilingualism *per se*, including executive control limitations in handling two languages in real time.

In order to fully acquire syntax-pragmatics interface constructions, L2 learners must discover the form-function mappings that are typical of L2, namely, mappings between syntactic forms and pragmatic functions (Callies, 2009, p. 87; Zhang and Jiang, 2021). Such mappings do not necessarily occur in a one-to-one fashion in that a pragmatic notion can be expressed by more than one syntactic construction (one-to-more mapping), which is more difficult to learn than a one-to-one mapping. Additionally, as syntax-pragmatics interface constructions require the efficient integration of changing contextual information (Sorace, 2016), the acquisition of them needs to observe and evaluate broader context and their functions within that context (Slabakova, 2015). Therefore, L2 learners need to recognize whether possible word orders can be used interchangeably or are restricted to particular pragmatic contexts.

## English existential constructions at the syntax–pragmatics interface

As a pragmatic mean to reorganize the information structure of utterances, English existential constructions are used to introduce hearer-new information into the discourse, that is, their postverbal NPs must represent hearer-new information which refers to information that a speaker assumes unfamiliar to a hearer (Ward et al., 2002). In many cases, the presence of new information makes the existential pragmatically obligatory in that the corresponding non-existential is infelicitous, as shown in example (1). This canonical word order normally cannot introduce new information into the discourse in an out-of-blue context, but the existential construction enables new information to move to the postverbal position, which conforms to the “given before new” information principle.

(1) # A hole is in my jacket.
There’s a hole in my jacket.

On the contrary, if postverbal NPs represent hearer-old information, existential constructions will be infelicitous, while the corresponding canonical word order is felicitous (Birner and Ward, 1998, p. 103–104). As in example (2), when answering question A, “the dog” in sentence B1 represents hearer-old information as it has been mentioned in question A, rendering this existential construction infelicitous, but the corresponding canonical word order in sentence B2 is felicitous.

(2) A: Have you seen the dog or the cat around?
B1: Not lately. #There’s the dog running loose somewhere in the neighborhood.
B2: Not lately. The dog is running loose somewhere in the neighborhood.

In addition, there’s no “definiteness restriction” on a postverbal NP in existential constructions, that is, definite NPs are admissible provided they represent hearer-new information (Ward et al., 2002). As shown in example (3), in (3)a, sentence B is a felicitous answer to question A1 in that “my father” represents hearer-new information, but it is an infelicitous answer to question A2 because “my father” has the same referent as “your father” in question A2. Likewise, in (3)b, definite NPs “Harry” and “Mrs. Jones” are also felicitous in sentence B.

(3) a. A1: Who is in the queue?
A2: Where is your father?
B: There is my father in the queue.
b. A: Is there anyone coming to dinner?
B: Yes, there’s Harry and also there’s Mrs. Jones.

Regarding the counterpart to English existential constructions in Chinese, it is existential *you*-construction that is the closest counterpart both pragmatically and syntactically (Huang, 1987). Specifically, existential *you*-construction is also used to introduce new information into the discourse, and “you” is in front of NPs just like “there be.”

**Figure 1:**
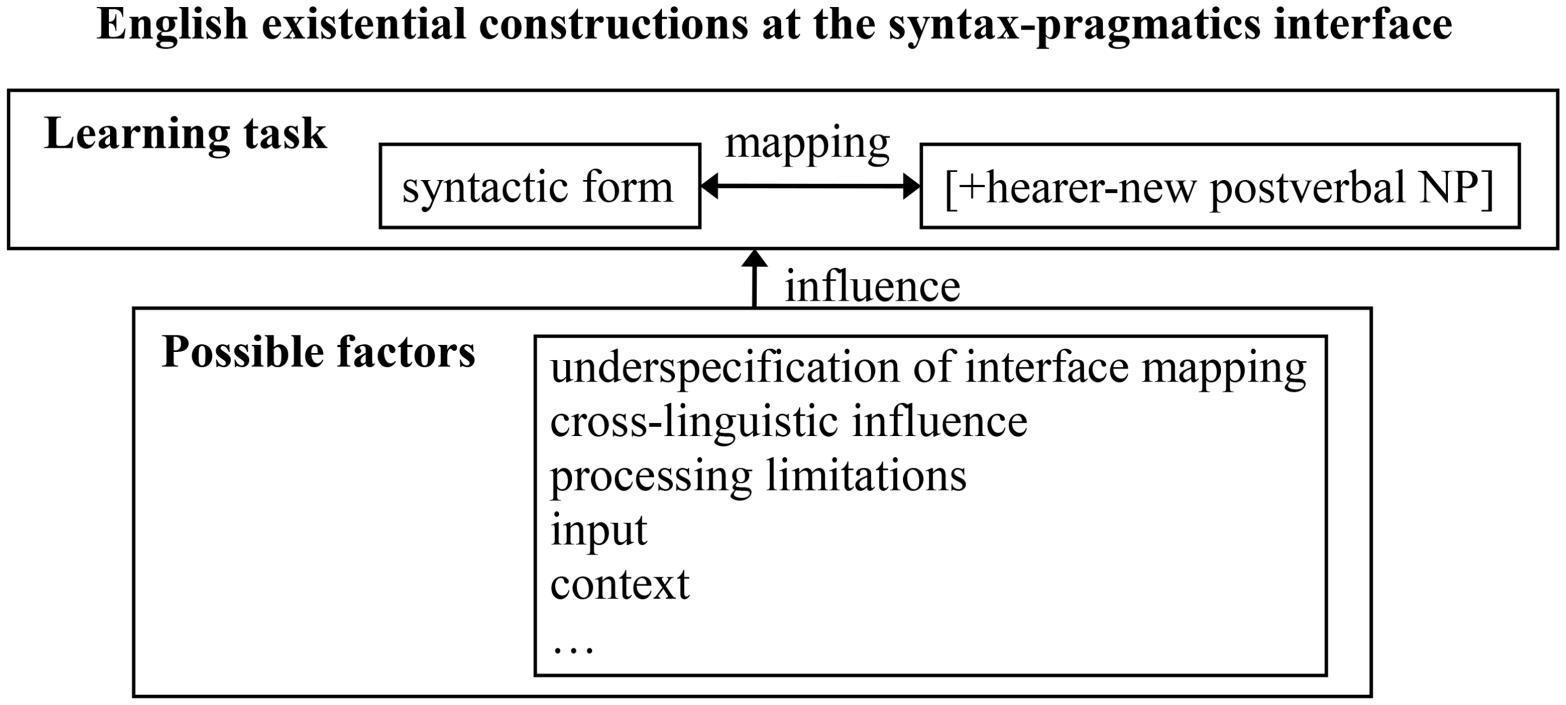
English existential constructions at the syntax–pragmatics interface.

In summary, the acquisition of English existential constructions requires L2 learners to discover their form-function mapping: postverbal NPs must be hearer-new, no matter they are indefinite NPs or definite NPs. For brevity, such contexts are represented as “new information contexts” in this study. Figure 1 shows the learning task of English existential constructions at the syntax-pragmatics interface and possible factors influencing it illustrated in section “Interface Hypothesis.”

## Research methodology

In this section, information regarding participants, test instruments, procedures and data analysis will be presented in detail in order to explain the research design.

### participants

This study included 320 participants. Among the total sample, 300 participants were Chinese-speaking learners of English as the experimental group, and 20 were native speakers of English as the control group. These Chinese learners are students majoring in English at a university in China. The native speakers are foreign teachers and students at this university. To select learners of three proficiency groups, this study adopts purposive sampling from first-year undergraduates, postgraduates and Ph.D. students based on their English scores. The sampling standard is as follows. The first-year undergraduates whose score in National Matriculation English Test (NMET) is between 100 and 140 are sampled as the elementary group. It is a nationwide unified exam for high school students for admissions to general colleges, whose full score is 150. As for the postgraduates and Ph.D. students, those who score between 60 and 65 in Test for English Majors-Grade 8 (TEM-8) are sampled as the intermediate group, while those who score above 70 are sampled as the advanced group. Each group selects 100 samples. Moreover, a Mann–Whitney U test further showed a significant difference in the scores of the intermediate and advanced groups (*Z* = −12.266, *p* < 0.001). Information of each group is given in Table 1.

**Table 1:** Information of each group.

Groups	Samples	Sampling standard	Mean scores	Number
ELE	First-year undergraduates	100 ≤ score of NMET≤140	123.5	100
INT	Postgraduates, Ph.D. students	60 ≤ score of TEM-8 ≤ 65	63.8	100
AD	Postgraduates, Ph.D. students	Score of TEM-8 ≥ 70	72.4	100
NG	Foreign teachers and students			20

ELE, Elementary group; INT, Intermediate group; AD, Advanced group; NG, Native group.

### Test instruments

All the participants were required to do two tests in total. A picture description test is for testing the production of existential constructions in new information contexts. A context-matching test aims to examine the comprehension of the syntax-pragmatics interface of existential constructions. Follow-up semi-structured interviews are conducted within a part of participants to capture the thought processes of Chinese learners and enrich the quantitative results.

#### Picture description test

The picture description test is adapted from the “Headlines” test employed in Belletti et al. (2007), by which participants report some event in a picture eliciting an all-focus context, using the sentence fragments and starting by saying “have you heard that…” However, contexts in which postverbal NPs represent hearer-new information in our test must be elicited by specific questions, and sentence-initial constituents are not fixed, but up to participants.

Totally, this test consists of 20 test items, including 10 experimental items and 10 fillers. Each experimental item gives a picture representing locations of some objects, followed by a particular question eliciting a “new information context,” such as What’s + PP? (4). Such questions can create contexts in which the use of existential constructions becomes more natural and likely. However, this is not to say that existential constructions are the only acceptable answer. We just expect that participants will produce these constructions at least in some of these “new information contexts.” In order to avoid elliptical answers, a series of sentence fragments in random order are given after each question. The participants are asked to answer the question by using all the fragments in one sentence.

(4) What’s beside the street? (hotel, beside the street, restaurant)

#### Context-matching test

The context-matching test is adopted from Lozano (2006), but we add a filler among the responses in each context. Overall, this test consists of 24 test items, including 12 experimental items and 12 fillers. Each test item provides a context by a short dialog in the form of question-answer pair. The experimental items involve three sets of variables: contexts ([+new information context], [+old information context]), word orders (existential construction, canonical word order), and “definiteness” of postverbal NPs in [+new information context] ([−definite NP], [+definite NP]). Each variable value of context and definiteness has four test items.

In [+new information context], in order to establish a context felicitous to move backward a NP, a question inducing new information NP in the response is asked, such as Who’s + PP, as in example (5) and (6). Example (5) exemplifies an indefinite NP, and example (6) illustrates a definite NP. In [+old information context], a question that triggers old information NP in the response is asked, such as Where’s + NP, as in example (7). A question is followed by three responses in a randomized order, namely, an existential construction, a canonical word order and filler. All test sentences are grammatically correct. The participants are required to judge the appropriateness of each test sentence. In this test, −2 stands for *completely inappropriate*, −1 for *possibly inappropriate*, 0 for *not sure*, +1 for *possibly appropriate*, +2 for *completely appropriate*.

(5) Lisa works during the day, and her daughter stays at home alone today. When Lisa comes back home, she says to her daughter: “Dear Annie, anything interesting happens in the neighborhood today?”
  Annie answers: _____.
  A. A funny-looking dog is in the neighborhood.
  B. I do not like staying at home alone.
  C. There is a funny-looking dog in the neighborhood.
(6) Robert and his parents are at Disneyland. He wants to go quickly because many tourists are waiting in line in front of the venue. While his dad says they do not need to hurry as someone has been in the queue already. Robert asks: “Who’s in the queue?”
  His dad answers: _____.
  A. My friend is in the queue.
  B. It’s polite to wait in the queue.
  C. There is my friend in the queue.
(7) Tina and Peter are getting married next month. Tina is making a video call with her sister now. Her sister congratulates on her marriage, and wants to have a look at their wedding photos. Tina asks Peter: “Where are the wedding photos?”
  Peter answers: _____.
  A. The wedding photos are in the drawer.
  B. The wedding photos are quite beautiful.
  C. There are the wedding photos in the drawer.

#### Semi-structured interviews

Semi-structured interviews are expected to let interviewees analyze the reasons of certain acquisitional phenomena in order to explain non-native performance at the syntax-pragmatics interface. The questions in the interviews are guided by the possible factors in Figure 1.

Regarding the picture description test, the questions center on: (1) What method did you adopt when writing this sentence? (2) What do you think of the degree of difficulty in writing out this sentence? (3) Can you come up with other sentences to answer this question? Why did not you use this sentence when doing the test?

With respect to the context matching test, the questions mainly involve: (1) Compared with the canonical word order, what’s the pragmatic function of existential constructions? (2) Do you think the use of existential constructions is constrained by contexts? Why? (3) In existential constructions, how often do you encounter and use indefinite and definite NPs?

### Procedures

At first, we designed a questionnaire of two tests. Instructions for the tests are given in the participants’ native languages to ensure a full understanding of the instructions. The test sentences are from the examples in previous studies on existential constructions, which are modified on the basis of Chinese learners’ English level and cultural background. The experimental and filler items are presented in a randomized order, with the provision that no more than two items from the same variable appears consecutively. Secondly, a pilot study was conducted by randomly selecting ten learners from each L2 group and five native speakers to examine the test items. Accordingly, the items were modified. Thirdly, the formal test was implemented in the form of a timed questionnaire *via* an online questionnaire system called Wenjuanwang. The questionnaires were sent to participants *via* a social app named WeChat. Participants were required to complete it independently without referring to dictionaries or other people. After collecting the questionnaires, we checked for any outliers and incomplete responses; altogether, 320 valid questionnaires were retained. Ultimately, semi-structured interviews were implemented. Ten participants from each L2 group were selected on the basis of rigorous standards. They must be equipped with specialized English knowledge, interested in this experiment and willing to cooperate. Each interview was carried out in Putonghua for about half an hour and audio-recorded with participants’ permission. All participants were informed of the research purposes and assured that their personal information would remain confidential. These procedures guaranteed the trustworthiness of the study.

### Data analysis

All questionnaire data were recorded and analyzed using SPSS 26.0. We first tested the reliability and validity of the context-matching test. Results showed that Alpha is 0.864 and KMO is 0.809, which indicated this test has a high reliability and validity. Then questionnaire data of the picture description test were codified. An experimental item has a binary outcome—absence or presence of existential constructions. A value of “1” is given for each existential construction that participants produced, and a value of “0” is given for other responses. Ultimately, descriptive analyses were conducted using comparing means, and statistical analyses were conducted using generalized linear mixed model (GLMM). We opted for this model over repeated-measures ANOVAs because a variable has several test items contributing to two random effects: performance variability between individuals and individual performance variability across items. This model can consider whether variability on participants and items affects the results, thus having a high statistical power. In each model, the independent variables are modeled as fixed effects, such as group, context, word order and definiteness. Participants and items are modeled as random effects. The dependent variables are participants’ productions of constructions and choices of number on a Likert scale.

The interview data were first transcribed into texts. After 2 weeks, the researchers transcribed them again. The consistency of transcriptions turns out to be 100%. Then the texts were analyzed and generalized carefully in order to discover and classify the factors involved in the interviews.

## Results

In this section, the first part provides experimental results, including production results in the picture description test and comprehension results in the context-matching test, and the second part presents qualitative data related to factors for non-native performance in production and comprehension.

### Experimental results

#### Production results

Table 2 shows the production results of existential constructions in each group. L2 groups occupy much higher percentages (80.8, 75.3, 66.5%) than the native group (63.5%). The minimum and maximum numbers in the elementary group (6, 10) are higher than those in the native group (5, 8). These results reveal that L2 groups overproduce existential constructions in new information contexts compared with the native group, but they produce these constructions fewer and fewer with the improvement of proficiency. Similar result was found in Zhang’s (2016) study. She found the frequency of existential constructions in TEM-4 Oral Corpus was much higher than that in TEM-8 Oral Corpus, which showed the higher proficiency of Chinese learners were, the fewer existential constructions they used.

**Table 2:** Production results of existential constructions.

Group	Total number	Total percentage	Means	Standard error	Min.	Max.
ELE	808	80.8%	8.08	1.1849	6	10
INT	753	75.3%	7.53	0.9114	5	9
AD	665	66.5%	6.65	0.8636	5	8
NG	127	63.5%	6.35	0.9459	5	8

To explain this phenomenon explicitly, we list some typical output of participants in the experimental item shown in section “Picture description test**”** (see Example 8). The elementary group mostly produces simple existential constructions (e.g., ELE 18), and occasionally produces simple canonical word orders (e.g., ELE 37) and locative inversion (e.g., 62). The intermediate and advanced groups tend to produce complex existential constructions (e.g., INT 59, AD 26) and simple locative inversion. Some advanced learners can produce complex locative inversion (e.g., AD 45). In contrast, the native group mostly uses existential constructions and locative inversion (e.g., NG 10, NG 2). It can be seen that the elementary group relies too much on existential constructions, and the advanced group basically can use existential constructions and locative inversion flexibly.

(8) There is a hotel and a restaurant beside the street (ELE 18)
Hotel and restaurant are besides the street (ELE 37).
Beside the street are the hotel and restaurant (ELE 62).
There are a hotel and a restaurant standing beside the street (INT 59).
Although there is a hotel and a restaurant behind this street, there are some families (AD 26).
Beside the street is a hotel as well as a restaurant (AD 45).
There’s a hotel and a restaurant beside the street (NG10).
Beside the street are a hotel and a restaurant (NG 2).

As shown in Table 3, the main effect of group is significant (*p* < 0.001), which reveals that group has a significant influence on the production of existential constructions. The between-group comparisons show there is a significant difference between the elementary and native group, and the intermediate and native group (*β* = −0.831, SE = 0.1687, *t* = −4.924, *p* < 0.001; *β* = −0.623, SE = 0.1665, *t* = −3.744, *p* < 0.001), but there is no significant difference between the advanced and native group (*β* = −0.209, SE = 0.1634, *t* = −1.281, *p* = 0.200). This suggests that the production of existential constructions reaches a native-like level until the advanced group. The between-group comparisons within L2 groups show that there’s no significant difference between any of the two groups (*β* = −0.065, SE = 0.031, *t* = −1.097, *p* = 0.273; *β* = −0.143, SE = 0.075, *t* = −1.515, *p* = 0.130; *β* = −0.078, SE = 0.052, *t* = −1.552, *p* = 0.121). This indicates that the production of existential constructions within L2 groups exhibits a gradual decreasing trend.

**Table 3:** Results of GLMM.

Fixed effects				
Predictor	*F*	df1	df2	*p*
Intercept	16.175	3	3,196	<0.001
Group	16.175	3	3,196	<0.001

#### Comprehension results

**Figure 2:**
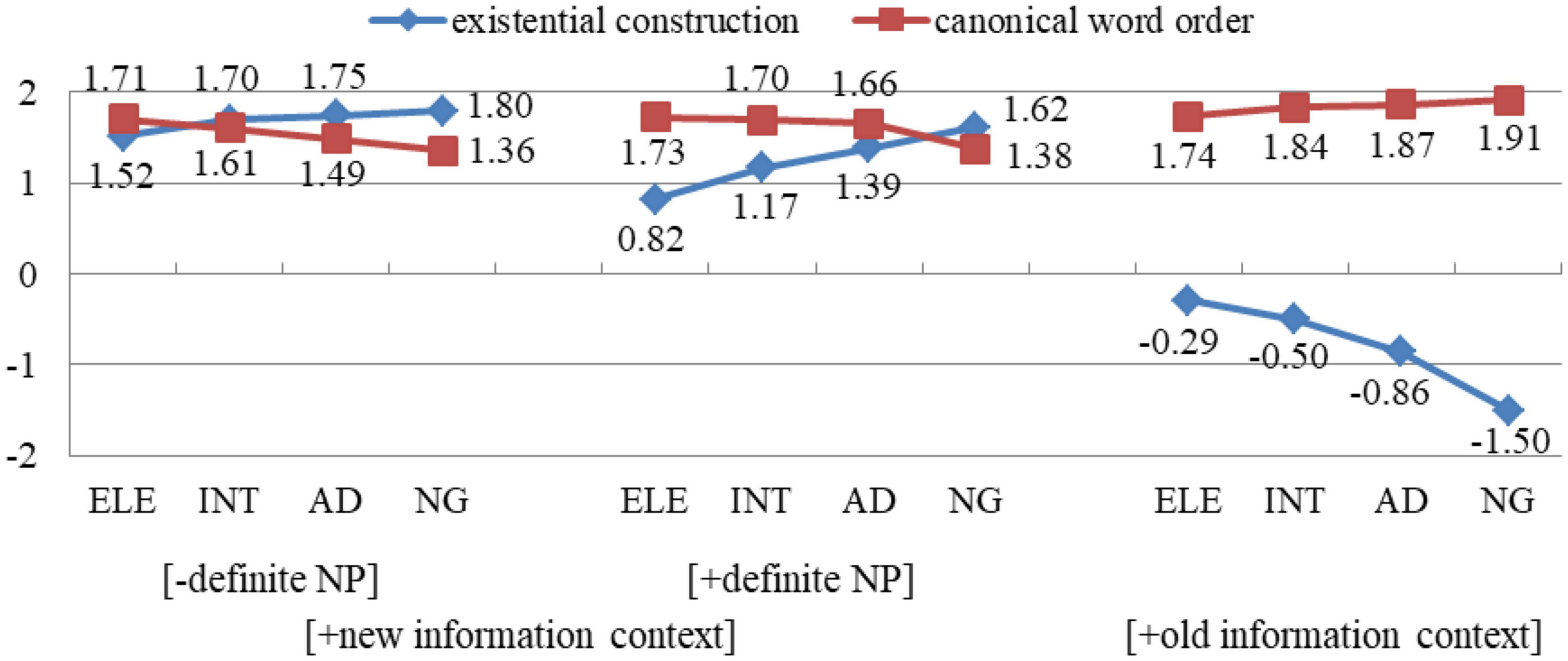
Mean scores of appropriateness of contexts in group.

Figure 2 provides data from the context-matching test. In [+new information context], each group accepts both existential constructions and the canonical word order. In comparison with the native group, L2 groups’ acceptance of existential constructions is lower, particularly those with definite NPs. In [+old information context], each group accepts the canonical word order and rejects existential constructions. However, L2 groups’ acceptance of existential constructions is much higher than that of the native group. From these results, we can see that L2 groups display different preference patterns for existential constructions from the native group. They accept appropriate existential constructions to a lower degree and accept inappropriate ones to a higher degree, but exhibit a developmental trend towards the native group.

In Table 4, the main effect of group, context and word order are all significant (*p* < 0.001, *p* < 0.001, *p* < 0.001), which demonstrates these variables all have a significant influence on the judgement of appropriateness. Between-group difference is significant between the elementary and native group (*β* = −0.068, SE = 0.5819, *t* = −8.153, *p* = 0.026), but it is insignificant between the intermediate and native group, and the advanced and native group (*β* = −0.113, SE = 0.2755, *t* = −2.373, *p* = 0.080; *β* = −0.104, SE = 0.0862, *t* = −1.390, *p* = 0.164). This reveals that the comprehension of the syntax-pragmatics interface has reached a native-like level by the intermediate stage. Paired comparisons show that only the elementary group’s acceptance of existential constructions in [+new information context] is significantly lower than the native group (*β* = 0.545, SE = 0.935, *t* = 12.003, *p* < 0.001). Regarding existential constructions in [+old information context], L2 groups show a significant higher acceptance compared with the native group (*β* = 1.202, SE = 0.716, *t* = 17.892, *p* < 0.001; *β* = 0.991, SE = 0.063, *t* = 15.138, *p* < 0.001; *β* = 0.640, SE = 0.046, *t* = 12.978, *p* < 0.001).

**Table 4:** Results of GLMM.

Fixed effects				
Predictor	*F*	df1	df2	*p*
Intercept	759.477	15	7,658	<0.001
Group	6.859	3	7,658	<0.001
Context	1985.778	1	7,658	<0.001
Word order	4927.351	1	7,658	<0.001
Group^*^Context	60.943	3	7,658	<0.001
Group^*^Word order	4.656	3	7,658	0.003
Context^*^Word order	2937.361	1	7,658	<0.001
Group^*^Context^*^Word order	115.405	3	7,658	<0.001

To explore the effect of definiteness on appropriate existential constructions, a GLMM is conducted and the results are shown in Table 5. The main effect of group, word order and definiteness are all significant (*p* < 0.001, *p* < 0.001, *p* < 0.001), which demonstrates these variables have a significant influence on the judgement of appropriateness. Paired comparisons show that the acceptance of existential constructions with indefinite and definite NPs is significantly different within each L2 group (*β* = 0.694, SE = 0.075, *t* = 13.980, *p* < 0.001; *β* = 0.533, SE = 0.070, *t* = 11.871, *p* < 0.001; *β* = 0.362, SE = 0.037, *t* = 4.283, *p* = 0.046), but is insignificant within the native group (*β* = 0.174, SE = 0.083, *t* = 1.914, *p* = 0.081). This reveals L2 groups display a rather low acceptance of existential constructions with definite NPs, while the native group accepts definite and indefinite NPs to a similar degree.

**Table 5:** Results of GLMM.

Fixed effects				
Predictor	*F*	df1	df2	*p*
Intercept	135.528	15	5,122	<0.001
Group	18.437	3	5,122	<0.001
Word order	202.268	1	5,122	<0.001
Definiteness	27.481	1	5,122	<0.001
Group^*^Word order	128.744	3	5,122	<0.001
Group^*^Definiteness	18.163	3	5,122	<0.001
Word order^*^Definiteness	402.729	1	5,122	<0.001
Group^*^Word order^*^Definiteness	5.515	3	5,122	0.001

### Qualitative data

#### Factors for non-native performance in production

In the interviews, when asked what strategies you adopted in writing this sentence, some interviewees responded that they first translated the question in test items into Chinese by sentences like “what does a place has,” and then described the pictures in Chinese sentences like “a place has…,” and finally translated them into English. That is to say, they transferred Chinese sentences into the process of producing English existential constructions. Some of their responses are shown in example (9).

(9) At first, I first described what objects the picture has in Chinese, and accordingly translated it into English. In my opinion, I can write this sentence out more easily after saying clearly what objects are in a certain place. (ELE 1)

Most of the sentences were written directly according to the pictures, while some were first expressed in Chinese and then translated. (AD 10)

When asked what other sentences they can think up to answer the questions, most of the interviewees answered locative inversion. Then when asked why they did not use this construction in the test, they responded that it is more difficult than existential constructions, and is rarely seen in English textbooks and used in daily conversations. On the contrary, existential constructions are the most frequently used constructions to express something in someplace. Consequently, their first reaction was to use existential constructions when doing the test. Some of their responses are shown in example (10).

(10) In my view, this is because that locative inversion is ordinarily seldom used, so it cannot be thought up when doing the test. No matter in English textbooks or oral conversations, “there be” sentences are almost always used to express something in someplace. (INT 9)

Locative inversion is more difficult than “there be” sentences, and it seldom occurs in English books, and even less in oral English. As a result, when expressing something in someplace, I cannot come up with locative inversion but “there be” sentences at once. (AD 1)

When asked about the degree of difficulty in writing out a sentence, the interviewees who wrote out existential constructions all responded that it was quite easy, and even those who wrote out canonical word orders responded that they actually could come up with existential constructions immediately. Therefore, processing limitations play no part in the production of these constructions. Some of their responses are shown in example (11).

(11) Actually, I can come up with using “there be” sentences to express something in someplace if I paid a little attention. Maybe I did not think when doing the test, and I was a little anxious. (ELE 10)

It’s very easy because I’ve been exposed to this structure since I was at the elementary school, and the teachers have taught its usage many times. (INT 5)

Consequently, the production of existential constructions is impacted by L1 influence and input frequency, but not processing limitations.

#### Factors for non-native performance in comprehension

When asked about the pragmatic function of existential constructions, the interviewees’ answers revealed that they had a one-sided picture of it. They only knew these constructions are used for expressing something in someplace, but did not mention they are used to introduce hearer-new information into the discourse or the information status of postverbal NPs. In a word, they have not discovered the form-function mapping of English existential constructions. Some of their responses are shown in example (12).

(12) The function of “there be” sentences is to express something in someplace. It can be said that they are the earliest learnt and easiest structures, so the function of them is very familiar to me. (ELE 4)

The function of this structure is to express something in someplace, which was clearly taught and probably known to everyone. (AD 3)

When asked whether the use of existential constructions is constrained by contexts, the interviewees gave a negative answer. They held that these constructions can be used whenever they want to express something in someplace. This is because that they have never learned the context constraint on existential constructions in English class or discovered this in use. Some of their responses are shown in example (13).

(13) There’s no context constraint. I learned this structure at the beginning of learning English, and I’ve never learned that it is restricted by any constraint, but only learned that it expresses something in someplace. (INT 2)

This structure is not constrained by contexts. I’ve never learned knowledge of this aspect, or thought about this issue when using this structure. (AD 4)

When asked about the exposure and usage frequency of indefinite and definite NPs in existential constructions, the interviewees responded that they were exposed to and used indefinite NPs quite frequently in sentences such as “there is a + singular noun” and “there are some/many + plural noun.” However, the exposure and usage frequency of definite NPs was rather low, such as person names, possessive pronouns and a definite article followed by nouns. Some of their responses are shown in example (14).

(14) It seems that this is the first time that I encountered the verb “be” followed by person names like Mary, Sue and Sam. I feel that I’ve never seen this before. What I’ve seen is “there is a” and “there are some.” Of course, I’ve never used person names after the verb “be” (ELE 2).

I’m seldom exposed to and use such possessive pronouns followed by nouns. “There be” sentences are always followed by an indefinite article and a singular noun, or nouns like many or some followed by a plural noun. (INT 4).

In conclusion, the comprehension of English existential constructions at the syntax-pragmatics interface is influenced by underspecification of form-function mapping, contexts and input frequency.

## Discussion

Within the framework of the IH, this section elaborates on the acquisitional features of English existential constructions at the syntax-pragmatics interface in production and comprehension, as well as factors for non-native performance at this interface.

### Discussion on acquisitional features of existential constructions

#### Discussion on acquisitional features in production

Our study finds that the elementary and intermediate groups overuse existential constructions to introduce existents as new information, and the advanced group basically can use different syntactic means to fulfill this function. This result is consistent with some previous studies (Palacios-Martínez and Martínez-Insua, 2006; Huang and He, 2007; Yang and Li, 2012; Zhang, 2016; Liu and Wang, 2020; Qin and Ding, 2021). Despite discrepancies in test instruments, L1-L2 pairings and L2 proficiency, these studies universally found L2 learners overused existential constructions. For instance, Huang and He (2007) found that most Chinese college students always used “There+be+NP + PP” structure to express something or somebody in someplace in a translation test. Zhang (2016) also found Chinese learners overused English existential constructions, especially those with simple and basic forms. Qin and Ding (2021) found the frequency of “there+be+NP” structure in Chinese learners’ corpus (11.3%) was higher than that in English native speakers’ corpus (9.6%), but the difference is insignificant.

However, Gong (2019) yielded a different result. From the perspective of topic/subject prominence of language typology, this study found that Chinese learners underused English existential constructions, and failed to use them in situations where they should be used. In our view, the contradictory results are due to distinction in both research perspectives and methods. This study was from a language typology perspective and aimed to investigate the transfer of Chinese topic-prominent features into English with subject-prominent features. Accordingly, this study adopted a translation and a story-retelling test including Chinese topic-prominent constructions, which contributed to Chinese learners more probably to transfer Chinese topic-prominence. This resulted in the underuse of English existential constructions. Instead of adopting this perspective, other studies aimed to examine the properties in the production of English existential constructions. Correspondingly, they employed natural learner corpus or translation tests involving sentences expressing something in someplace, which resulted in Chinese learners more likely to produce English existential constructions.

#### Discussion on acquisitional features in comprehension

This study finds that in [+new information context], L2 groups attribute a weaker level of appropriateness to appropriate existential constructions, particularly the elementary group. In [+old information context], the acceptance of inappropriate existential constructions is too high within L2 groups. Our finding is consistent with Teixeira (2020). He found that Portuguese and French learners of English failed to discriminate appropriate and inappropriate existential constructions, and over 50% participants accepted or rejected these constructions in all the contexts, exhibiting optionality.

Conversely, some previous studies reported different results (Lozano and Mendikoetxea, 2008, 2010; Agathopoulou, 2014; Mendikoetxea and Lozano, 2018). These studies suggested that L2 learners with different L1 backgrounds were aware of the syntax-pragmatics interface constraint on existential constructions and did not use them in inappropriate contexts. A likely explanation for the contradictory results may be related to different test instruments. The second kind of results mainly came from learner corpus merely involving production data, which gives rise to avoidance. That is, when L2 learners are not sure whether existential constructions are appropriate in a certain context, they may avoid using it. Therefore, these studies could not safely assert that learners attained native-like syntax-pragmatics competence, as it may result from avoidance. While this study adopted a context matching test, and Teixeira (2020) adopted a syntactic priming and acceptability test, these tests can investigate more accurately whether participants are actually equipped with implicit knowledge of the syntax-pragmatics interface.

Regarding definiteness, this study finds that L2 groups’ acceptance of existential constructions with definite NPs is rather lower than those with indefinite NPs. A similar result was found in Huang and He (2007), who found Chinese learners hardly ever used English existential constructions with definite NPs and attributed a low acceptance to them. However, other studies showed contradictory results: L2 English learners showed a native-like judgment on definite and indefinite NPs in existential constructions (White, 2008; White et al., 2012). In our view, the distinct results between the above studies and our study result from differences in three aspects: the research content and setting of test items, L1-L2 pairings and participants’ English learning backgrounds. Firstly, these prior studies held that there’s definiteness restriction on existential constructions, that is, indefinite NPs are allowed while definite NPs are precluded. Accordingly, the setting of test items was existential constructions with indefinite NPs in appropriate contexts, and those with definite NPs in inappropriate contexts. While our study adopts the viewpoint that there’s no definiteness restriction, and thus both indefinite and definite NPs are in appropriate contexts. In fact, definite NPs are not correspondent in prior studies and our study. Secondly, the L1s in White et al. (2012) were Turkish and Russian observing a definiteness restriction similar to English, which facilitated the acquisition of English. Nonetheless, this is not the case for Chinese which lacks an article system. Finally, Chinese participants in White (2008) were living in Canada and the average length of residence was two years. They were exposed to more natural and adequate English than Chinese participants in foreign classroom settings in this study. It’s easier for them to acquire the definiteness restriction.

### Discussion on factors for non-native performance

#### Discussion on factors for non-native performance in production

Combined with the qualitative data, the overproduction of existential constructions is due to L1 negative transfer and input frequency, which will be elaborated in turn in the following part.

Firstly, according to the IH, cross-linguistic influence has an impact on the acquisition of the syntax-pragmatics interface in representations and parsing. With language experiences of Chinese, Chinese learners’ language system and learning mechanism have been accustomed to Chinese input, and it will be inevitably activated which will interfere with the process of learning English. Due to the close correspondence between Chinese existential *you*-construction and English *there*-construction mentioned in section “English existential constructions at the syntax-pragmatics interface”, this will facilitate the production of English existential constructions within Chinese learners. As reflected in the interviews, Chinese learners considered existential *you*-constructions as the counterpart of English “there be” sentences. They transferred Chinese knowledge into the production of English existential constructions, and used them to express the meanings construed in Chinese. As a result, Chinese learners are more likely to use existential constructions to introduce new information. With the improvement of English proficiency, the advanced learners gradually get rid of Chinese interference, and are equipped with the ability to use English knowledge in production.

Secondly, the input frequency of a structure is bound to have an effect on the speed and accuracy with which it is processed. As is evident from the interviews, Chinese learners have studied existential constructions and done lots of exercises since the initial stage of English learning. Furthermore, a previous study showed that among the sentences expressing something in someplace, existential constructions occupy up to 98 and 99%, respectively, in junior and senior high school and college English textbooks (Huang and He, 2007). Obviously, Chinese learners are exposed to adequate existential constructions. With increasing exposure, these constructions will become more and more stable, and eventually the mapping between their syntactic forms and pragmatic function will be established. As a result, this function can hardly activate other syntactic forms, like locative inversion. Therefore, the usage of existential constructions is entrenched and their processing is automated within Chinese learners. In online production, learners tend to choose a construction with high input frequency, which is more easily to access and produce. For this reason, the elementary and intermediate learners overproduce existential constructions.

Similarly, the mastery of locative inversion also requires a large amount of input, but this construction rarely occurs in English classes and textbooks according to what the interviewees said. Its low input frequency impedes its processing. Consequently, elementary and intermediate Chinese learners fail to come up with locative inversion immediately in production. With increasing exposure to English, advanced learners gradually notice that locative inversion has the same pragmatic function as existential constructions, and thus tend to produce locative inversion instead of relying solely on existential constructions.

#### Discussion on factors for non-native performance in comprehension

Combined with the qualitative data, L2 groups’ different preference patterns for existential constructions from the native group are caused by underspecification of form-function mapping, contexts and input frequency. These factors will be elaborated one by one in the following part.

Firstly, as pointed out in section “English existential constructions at the syntax-pragmatics interface,” the acquisition of English existential constructions involves learning the mapping between syntactic forms and hearer-new NP. The difficulty lies in that hearer-new NP, involving both indefinite and definite NPs, can be expressed by means of either existential constructions or their corresponding canonical word order. However, Chinese learners did not know the pragmatic function of introducing hearer-new information, and certainly failed to master the mapping rule of existential constructions. What’s worse, the canonical word order is more common and unmarked, leading to learners mapping the pragmatic function onto this word order. It’s difficult for them to notice that existential constructions with definite NPs also perform this function. Consequently, they display a weak preference for these constructions in [+new information context], but a strong preference for the canonical word order.

Secondly, the acquisition of syntax-pragmatics interface structures needs to observe and evaluate broader context and their functions within that context. Concerning existential constructions, Chinese learners need to identify whether they are used universally or restricted to particular pragmatic contexts, and recognize these contexts as new information contexts. Nonetheless, as the real-world and discourse contexts of linguistic utterances are extremely rich, learners will find it difficult, if not impossible, to identify the specific contexts that license use of existential constructions. Furthermore, learners are not provided with negative evidence that these constructions are infelicitous in old information contexts. As a result, Chinese learners fail to identify the context constraint on existential constructions and consider them not constrained by contexts, leading to over-accepting inappropriate existential constructions in old information contexts.

Thirdly, the effect of input frequency has mentioned earlier. The interviewees demonstrated that they have been seldom exposed to existential constructions with definite NPs. In fact, among all the existential constructions in junior and senior high and college English textbooks, those with definite NPs merely take up 1 and 4%, respectively (Huang and He, 2007). In such scarce input, it’s difficult for Chinese learners to discover the usage pattern of definite NPs in existential constructions. Consequently, they attribute a low acceptance to these constructions. On the contrary, indefinite NPs are easier to learn because of their adequate input. Learners have been exposed to many sentences like “there is a…” and “there are some/many…” from which they can induce that existential constructions occur with indefinite NPs.

It’s noteworthy that the intermediate group’s comprehension of the syntax-pragmatics interface reaches a native-like level, but the production of existential constructions does not. This is attributable to two reasons. In one aspect, production tests bring a lot more cognitive burden to participants than comprehension tests. In another, in online production, the real-time integration of syntactic and pragmatic information is more difficult, which demands participants employing cognitive resources to integrate information effectively at any time. Similar results were found in some earlier studies (Sequeros-Valle et al., 2020; Teixeira, 2020). For instance, Sequeros-Valle et al. (2020) investigated the comprehension and production of pragmatic restrictions in clitic-doubled left dislocation within English learners of Spanish. They found that learners showed native-like knowledge in an acceptability test, but non-native performance in a timed production test.

In conclusion, Chinese learners’ overproduction of existential constructions results from L1 negative transfer and input frequency. They display different preference patterns for these constructions from native speakers, which attributes to underspecification of form-function mapping, contexts and input frequency.

## Conclusion

From the perspective of the IH, this study investigated acquisitional features of English existential constructions by Chinese learners of three proficiency levels, and explored the factors for non-native performance. It was found that the acquisition of the syntax-pragmatics interface by Chinese learners was rather delayed and displayed unbalance. Specifically, the production of existential constructions reached a native-like level until the advanced stage, while their comprehension reached this level by the intermediate stage. The analysis indicated that the overproduction of existential constructions resulted from L1 negative transfer and input frequency, and the inappropriate preference pattern for them attributed to underspecification of form-function mapping, contexts and input frequency.

In order to improve Chinese learners’ syntax-pragmatics competence in English, classroom instruction can be refined by regulating the factors that influence the acquisition of this interface. Firstly, explicit instruction in the form-function mapping rule of existential constructions should be provided for learners, including the pragmatic function, the concept of new information, and syntactic forms and pragmatic functions being not always in a one-to-one relation. In the meanwhile, teachers should help learners construct the form-function mapping rule. This explicit learning can activate learners’ attention to pragmatic functions, improve their awareness of syntax-pragmatics and eventually improve pragmatic appropriateness. Secondly, the input frequency of existential constructions with definite NPs in appropriate contexts should be increased. This is because that adequate linguistic experiences help to facilitate the establishment of the context-form pairing and the development of contextual knowledge. Thirdly, teachers should give a detailed comparison of the similarities and differences in the syntactic means of information packaging in Chinese and English in order to reduce L1 interference. Finally, learners should be trained to comprehend and produce through practice in order to internalize knowledge of the syntax-pragmatics interface and access and integrate this interface more automatically. Gradually, they will attain native-like competence in both representations and production.

This study makes several noteworthy contributions to L2 research and teaching. First, these findings help to broaden our understanding of acquisitional features of the syntax-pragmatics interface, and reveal more deeply the role of this interface in the construction of English and in non-native performance. Second, these findings lend support to the IH. Notably, difficulties at the syntax-pragmatics interface are related to specific L1-L2 pairings. Meanwhile, they also enhance explanatory power in the illustration of interface vulnerability by adopting the semi-structured interviews, and some of the five factors have been verified as important factors in the acquisition of this interface. Finally, these findings provide productive insight into the teaching and learning of the syntax-pragmatics interface, which serves to decrease non-native performance fundamentally. For one thing, teachers can modify the teaching method of this interface in a targeted manner. For another, L2 learners can be provided with actual data resources in order to improve explicit knowledge.

Although this study endeavored to depict a comprehensive picture of L2 acquisition at the syntax-pragmatics interface, it had some limitations that suggest directions for future research. First, both the questionnaire and interview sample sizes were somewhat small in this study. It is necessary for future research to enlarge the research sample to involve Chinese learners at different levels to enrich the studies on L2 acquisition of the syntax-pragmatics interface. Second, while the main instruments in this study were offline timed tests, future studies are advised to utilize online tasks that record reaction times or ERPs to investigate the effect of processing limitations on the syntax-pragmatics interface predicted by the IH. Finally, researchers can further validate and explore factors for interface vulnerability through more qualitative methods like think-aloud to better understand the fundamental reason for the acquisition difficulty of this interface.

## Data availability statement

The raw data supporting the conclusions of this article will be made available by the authors, without undue reservation.

## Ethics statement

The studies involving human participants were reviewed and approved by Professor Committee of School of Foreign Languages, Northeast Normal University. Written informed consent to participate in this study was provided by the participants' legal guardian/next of kin.

## Author contributions

SJ was responsible for the conception and design of the study, data collection, data analysis and interpretation, writing, developing, and editing the manuscript. HZ contributed to the manuscript development and editing. All authors contributed to the article and approved the submitted version.

## Funding

This research was funded by National Office for Philosophy and Social Sciences of China (Grant no. 20BYY209) and Youth Team Founding of Northeast Normal University (Grant no. 2021QT004).

## Conflict of interest

The authors declare that the research was conducted in the absence of any commercial or financial relationships that could be construed as a potential conflict of interest.

## Publisher’s note

All claims expressed in this article are solely those of the authors and do not necessarily represent those of their affiliated organizations, or those of the publisher, the editors and the reviewers. Any product that may be evaluated in this article, or claim that may be made by its manufacturer, is not guaranteed or endorsed by the publisher.
